# Association between Breastfeeding Duration and Type of Birth Attendant

**DOI:** 10.1155/2018/7198513

**Published:** 2018-03-01

**Authors:** Jordyn T. Wallenborn, Saba W. Masho

**Affiliations:** School of Medicine, Division of Epidemiology, Department of Family Medicine and Population Health, Virginia Commonwealth University, 830 East Main Street, Suite 821, P.O. Box 980212, Richmond, VA, USA

## Abstract

**Introduction:**

Healthcare providers play an integral role in breastfeeding education and subsequent practices; however, the education and support provided to patients may differ by type of provider. The current study aims to evaluate the association between type of birth attendant and breastfeeding duration.

**Methods:**

Data from the prospective longitudinal study, Infant Feeding Practices Survey II, was analyzed. Breastfeeding duration and exclusive breastfeeding duration were defined using the American Academy of Pediatrics' national recommendations. Type of birth attendant was categorized into obstetricians, other physicians, and midwife or nurse midwife. If mothers received prenatal care from a different type of provider than the birth attendant, they were excluded from the analysis. Multinomial logistic regression was conducted to obtain crude and adjusted odds ratios and 95% confidence intervals.

**Results:**

Compared to mothers whose births were attended by an obstetrician, mothers with a family doctor or midwife were twice as likely to breastfeed at least six months. Similarly, mothers with a midwife birth attendant were three times as likely to exclusively breastfeed less than six months and six times more likely to exclusively breastfeed at least six months compared to those who had an obstetrician birth attendant.

**Conclusions:**

Findings from the current study highlight the importance of birth attendants in breastfeeding decisions. Interventions are needed to overcome barriers physicians encounter while providing breastfeeding support and education. However, this study is limited by several confounding factors that have not been controlled for as well as by the self-selection of the population.

## 1. Introduction

While the majority of births are attended by obstetricians, the proportion of midwife attended vaginal births in the United States reached an all-time high (11.4%) in 2009 [1]. However, these rates vary drastically by state. For example, certified nurse-midwifes attended almost a quarter (23.9%) of all births in New Mexico whereas they attended only 0.8% of births in Arkansas. Despite these variations, the prevalence of midwife attended births has increased in almost all states since 1990 [1].

Literature has shown that births attended by midwives have improved outcomes. A large study conducted in Canada reported that mothers with a home-birth attended by a midwife had a reduced risk of birth trauma and resuscitation at birth compared to mothers with a planned hospital birth attended by a physician [2]. Midwives have also been reported to manage the third stage of labor based on the patient's preference whereas physicians were more likely to actively manage this stage of labor [3].

Research has also shown that midwives have greater knowledge of breastfeeding benefits and higher self-confidence when managing breastfeeding problems [4]. For example, over half of certified nurse midwives report being “well” or “very well” prepared to assist breastfeeding women. Also, 9 out of 10 midwives reported encouraging mothers to breastfeed more if mothers were concerned about insufficient milk supply [4], a major reason women prematurely cease breastfeeding [5].

In contrast to favorable outcomes shown among midwives, literature provides insight into physicians deteriorating attitudes and commitment to breastfeeding support [6–8]. A study that assessed practicing obstetrics/gynecologists, pediatricians, and family member physicians reported a significant deficit in the knowledge of breastfeeding benefits and clinical management [7]. In fact, a survey of pediatricians showed that 20% made no breastfeeding recommendation, 13% recommended breastfeeding with formula, and 2% recommended formula feeding only [9].

Numerous studies have assessed healthcare professional's knowledge of breastfeeding outcomes and attitudes and commitment to providing breastfeeding support [4, 6–9]. However, knowledge may not directly translate to clinical practice. Healthcare professionals may not have the skills, expertise, or time to provide adequate breastfeeding support to mothers. Nevertheless, these attributes may differ between types of healthcare professional. To better understand the ability of healthcare providers in providing breastfeeding counseling, the current study aims to investigate the association between type of birth attendant and breastfeeding duration.

## 2. Materials and Methods

Data from the prospective longitudinal Infant Feeding Practices Survey (IFPS) II was analyzed. IFPS II was conducted by the Food and Drug Administration and Centers for Disease Control and Prevention from May 2005 through June 2007. Information on maternal and child health, infant feeding behaviors, and a mother's diet was collected through questionnaires and a short telephone interview. To be included in the study, mothers were at least 18 years old at the time of the prenatal survey, had a full-term or nearly full-term singleton infant, and had good maternal and child health at birth. Good maternal and child health at birth was defined as “neither the mother nor the infant could have a medical condition at birth that would affect feeding and that the infant had to have been born after at least 35 weeks' gestation, weigh at least 5 lb, be a singleton, and not have stayed in the intensive care for >3 days” [10]. Additional information on IFPS II methodology can be found elsewhere [10].

The current study also applied exclusion criteria. Mothers were excluded if women received prenatal care from a different type of provider and then the birth attendant or reported no healthcare provider at birth (*N* = 15) or another type of healthcare provider (*N* = 34) due to small numbers or if women had missing information on breastfeeding duration and healthcare provider at birth, leaving 2,979 women for analysis. This study was approved as exempt by the Virginia Commonwealth University Institutional Review Board.

The main outcome variable, breastfeeding duration, was categorized as “never breastfed,” “breastfed less than 6 months,” and “breastfed at least 6 months” which is consistent with national recommendations [11]. Breastfeeding duration was based on three self-report survey items: “Did you ever breastfeed this baby (or feed this baby your pumped milk)?” If mothers responded “yes,” they were asked “Have you completely stopped breastfeeding and pumping milk for your baby?” every month until breastfeeding cessation occurred. To establish duration of breastfeeding after cessation, participants were asked, “How old was your baby when you completely stopped breastfeeding and pumping milk?” If mothers were still breastfeeding at the time of the last interview (at 12 months postpartum) (*N* = 917), the following question was asked at the six-year follow-up: “How old was your 6-year-old when the following happened? He or she stopped being fed breast milk, including pumped breast milk.” The current study also investigated excusive breastfeeding, which was defined as an infant's consumption of “no other food or drink, not even water, except breast milk (including milk expressed or from a wet nurse) … but allows the infant to receive oral rehydration solution (ORS), drops and syrups (vitamins, minerals and medicines)” [12].

Birth attendant, the main exposure variable, was based on the question, “Which type of health professional was your birth attendant?” which was assessed during the neonatal questionnaire. Healthcare provider at birth was categorized as “obstetrician,” “family doctor, general practitioner, internist, or other physicians,” and “midwife or nurse midwife.”

A variety of factors that were available in the dataset were considered as potential confounders. Demographic factors included marital status (married; not married), maternal race (White; Black; Hispanic; others including Asian/pacific islander), maternal age (18–24 years; 25–29 years; 30–34 years; 35–45 years), maternal education (less than high school; high school graduate; 1–3 years of college; college graduate), income (less than $20,000; $20,000–49,999; at least $50,000), and prepregnancy body mass index (underweight (<18.5 kg/m^2^); normal weight (18.5–24.9 kg/m^2^); overweight (25.0–29.9 kg/m^2^); obese (30.0+ kg/m^2^)). Healthcare variables included prenatal participation in the Special Supplemental Nutrition Program from Women, Infants, and Children (WIC) program (yes; no), postnatal participation in WIC, health insurance (yes; no), and mode of delivery (vaginally, not induced; vaginally, induced; planned Cesarean section; unplanned or emergency Cesarean section). Other factors included breastfeeding intention (breastfeed only; formula feed; or combination) and smoking during pregnancy (yes; no).

Descriptive statistics with frequencies and percentages were calculated to assess the distribution of characteristics by type of healthcare provider at birth. Separate bivariate multinomial logistic regression models produced crude odds ratio (COR) and 95% confidence intervals (CI) to show factors associated with breastfeeding duration. Effect modification for breastfeeding intention, marital status, race/ethnicity, education, and mode of delivery were assessed; however, they were not statistically significant. Therefore, all factors were considered as potential confounders. Multicollinearity was tested between pre- and post-WIC utilization and did not reach a variance inflation factor (VIF) of 5. A final parsimonious model including all factors that resulted in at least a 10% change in the crude estimate was used to generate adjusted odds ratios (AOR) and 95% CI. All analyses were conducted using SAS version 9.4 statistical software (SAS, Cary, NC). Virginia Commonwealth University Institutional Review Board determined the current study to be exempt.

## 3. Results

The majority of study participants were married (79.8%) and non-Hispanic white (85.6%), had at least some college education (80.2%), had health insurance (95.6%), had a vaginal delivery (71.4%), and initiated breastfeeding (85.4%) (Table 1). Over three quarters (86.9%) of mothers had an obstetrician as their birth attendant while 5.6% had a family doctor, and 7.5% utilized a midwife (not shown in Tables 1–4). Approximately 1 in 12 (8.2%) mothers exclusively breastfed for six months. Among mothers who used a midwife for their birth, more women were aged 25–29 years (37.4%), were normal weight (52.9%), intended to breastfeed only (78.1%), and gave birth vaginally (93.4%). Bivariate analyses demonstrated significant associations between all demographic, reproductive, and lifestyle factors and breastfeeding duration, except health insurance (Table 2).

Compared to mothers with an obstetrician birth attendant, those with a midwife were twice as likely (crude odds ratio (COR) = 2.32; 95% CI = 1.22–4.42) to breastfeed less than six months and almost four times (COR = 3.99; 95% CI = 2.12–7.49) more likely to breastfed at least six months. After adjusting for marital status, education, race, income, age, mode of delivery, breastfeeding intention, and prenatal and postpartum WIC participation, mothers with a family doctor, general practitioner, internist, or other physicians were twice as likely to breastfeed at least six months (AOR = 2.04; 95% CI = 1.04–4.00) compared to those who had an obstetrician birth attendant. Similarly, mothers with a midwife birth attendant were more than twice as likely to breastfeed at least six months (AOR = 2.43; 95% CI = 1.12–5.25) compared to those who had an obstetrician birth attendant (Table 3).

Similar results were obtained when investigating exclusive breastfeeding duration. Compared to mothers with an obstetrician birth attendant, those with a midwife were four times (COR = 4.01; 95% CI = 2.13–7.55) more likely to exclusively breastfeed less than six months and almost ten times (COR = 9.96; 95% CI = 4.78–20.75) more likely to exclusively breastfed at least six months. After adjusting for marital status, income, age, mode of delivery, and prenatal and postpartum WIC participation, estimates remained significant but slightly attenuated. Mothers with a midwife birth attendant were more likely to exclusively breastfeed less than six months (AOR = 3.11; 95% CI = 1.62–5.98) and exclusively breastfeed at least six months (AOR = 6.65; 95% CI = 3.05–14.50) compared to those who had an obstetrician birth attendant. No association was found among women whose birth was attended by obstetrician and family doctor, general practitioner, internist, or other physicians (Table 4).

## 4. Discussion

The current study found a relationship between type of birth attendant and breastfeeding duration. Specifically, mothers whose births were attended by midwives were more likely to breastfeed six months or more compared to mothers whose births were attended by obstetricians. Similarly, mothers whose birth was attended by midwives were more likely to exclusively breastfeed a longer duration compared to mothers whose birth was attended by obstetricians.

While no research, to our knowledge, has investigated the relationship between birth attendant and breastfeeding duration, the findings can be explained by the clinical management practices healthcare professionals utilize during the postpartum period. For example, recent literature has demonstrated that nurse midwives are more attentive and have more control over the education mothers receive [13]. Due to constraints of the current healthcare system, physicians spend limited time with their patients [14]. This could lead to (1) a lack of support while staying in the hospital or (2) a provision of inadequate information about breastfeeding to patients, all of which has the potential to influence breastfeeding practices. For example, a prospective cohort study reported that appraisal of the breastfeeding experience while in the hospital was significantly associated with breastfeeding success, defined as the mother successfully breastfeeding the duration planned at the mother's initial estimate [15]. Because physicians' time with patients is limited, nurses play a major role in postpartum care and breastfeeding education. Furthermore, studies have shown that nurses directly impact breastfeeding success through emotional, informational, and tangible support [16]. In fact, nurses' breastfeeding knowledge was the best predictor of breastfeeding support [14]. However, deficits in nurse's knowledge, attitudes, and commitment to breastfeeding have been reported [14].

Strengths of this study include the longitudinal prospective study design which allows temporality to be established. IFPS II also utilized a standardized data collection protocol that minimizes the potential for information bias. Other strengths include the ability to account for breastfeeding factors such as breastfeeding intention that can potentially affect breastfeeding outcomes. Further, the definition of breastfeeding duration includes breastfeeding exclusivity, which measures in full compliance with national recommendations [11].

Despite the numerous strengths, this study is not without limitations. Because a consumer opinion panel was used to identify participants, the study population disproportionately represents women who are white, are of higher socioeconomic status, can read English, and have stable mailing addresses. Therefore, results are not generalizable. Breastfeeding duration is self-report and subject to social desirability bias which could bias results towards the null; however, self-report of breastfeeding duration has been demonstrated as a reliable measurement [17]. Potential confounding factors such as facility where mother gave birth, labor and delivery staffing at the hospital where the mother delivered, if the mother self-selected the type of birth attendant and if hospitals had initiatives or policies that increase breastfeeding success (e.g., Baby-Friendly hospital policies), self-efficacy, and alcohol use were not available and may have impacted the effect size. Current literature lacks information on midwifery in hospitals. Future research should investigate correlates of midwifery practice within hospitals. Further, researchers should investigate if midwives are more likely to assist “skin-to-skin care.” Lastly, literature surrounding midwife and physician breastfeeding education and support is outdated. Additional research is needed to understand current breastfeeding knowledge among healthcare providers.

## 5. Conclusions

A woman's birth attendant was found to be significantly associated with breastfeeding duration and exclusivity. Despite the numerous health benefits and potential to improve maternal and child health, women are not receiving adequate support to breastfeed the recommended six-month duration. This is evident through a recent report from the Centers for Disease Control and Prevention which stated that mothers may not be receiving the breastfeeding support needed from healthcare providers [18]. Future studies are needed to understand the reasons for the low rates of breastfeeding among mothers attended by physicians. Current literature lacks information on midwifery in hospitals. Future research should investigate correlates of midwifery practice within hospitals. Further, researchers should investigate if midwives are more likely to assist skin-to-skin care. Interventions are also needed to overcome barriers encountered by physicians. Moreover, providers should be aware of the impact they can have on women's breastfeeding practices.

## Figures and Tables

**Table 1:** Distribution of maternal characteristics by healthcare provider at birth.

Characteristic	Total	Obstetrician	Family or other MD	Midwife	Chi square
	Percent *N* = 2,651	Percent *N* = 2,304	Percent *N* = 149	Percent *N* = 198	(*p* value)
*Age*					**0.0046**
18–24 years	22.1	21.4	26.4	27.8	
25–29 years	33.7	33.4	33.8	37.4	
30–34 years	27.9	27.9	31.8	24.2	
35–45 years	16.3	17.3	8.1	10.6	
*Marital status*					0.3323
Not married	20.2	19.8	24.1	22.5	
*Maternal race*					**0.0477**
White, NH	85.6	84.8	90.9	90.8	
Black, NH	4.4	4.6	4.2	2.0	
Hispanic	5.8	6.1	4.9	3.6	
Others	4.2	4.5	0.0	3.6	
*Maternal education*					**0.001**
Less than high school	3.0	2.6	5.1	6.5	
High school	16.8	16.3	24.8	16.7	
1–3 years of college	40.0	39.8	41.6	40.9	
College graduate	40.2	41.3	28.5	36.0	
*Income*					**0.0004**
<$20,000	13.0	12.2	19.5	17.2	
$20,000–$49,999	42.4	41.6	49.0	46.0	
≥$50,000	44.6	46.1	31.5	36.9	
*Prepregnancy BMI*					**0.0286**
Underweight	4.8	4.8	3.4	6.2	
Normal weight	45.3	45.1	39.2	52.9	
Overweight	25.5	25.1	33.8	23.8	
Obese	24.4	25.1	23.7	17.1	
*Health insurance*					**0.0005**
No	4.4	3.8	7.4	9.1	
*Postnatal WIC*					**0.0153**
Yes	38.9	37.9	48.3	43.4	
*Prenatal WIC*					**0.0494**
Yes	28.8	27.9	34.9	33.8	
*Mode of delivery*					**<0.0001**
Vaginally, not induced	37.4	33.6	53.0	69.7	
Vaginally, induced	34.0	35.0	31.5	23.7	
Planned C-section	16.5	18.3	8.1	2.0	
Unplanned or emergency C-section	12.2	13.2	7.4	4.6	
*Breastfeeding duration (any)*					**<0.0001**
Never	14.6	15.5	14.1	5.6	
<6 months	44.1	44.8	43.6	37.4	
≥6 months	41.2	39.8	42.3	57.1	
*Breastfeeding duration (exclusive)*					**<0.0001**
Never	25.3	27.3	23.9	7.6	
<6 months	66.5	65.7	68.2	73.1	
≥6 months	8.2	7.0	8.0	19.3	
*Breastfeeding intention*					**<0.0001**
Breastfeed only	60.1	59.1	53.0	78.1	
Formula or combination	39.9	40.9	47.0	21.9	
*Smoked during pregnancy*					0.6304
Yes	9.6	9.4	10.7	11.2	

NH = non-Hispanic; WIC = women; infants and children; BMI = body mass index; C-section = Cesarean section. *Note*. Not all percentages sum to 100% due to rounding.

**Table 2:** Factors associated with breastfeeding duration.

Characteristic	Breastfed < 6 months	Breastfed ≥ 6 months
	OR (95% CI)	OR (95% CI)
*Age*		
18–24 years	**1.45 (1.07–1.96)**	**0.41 (0.29–0.57)**
25–29 years	**1.77 (1.30–2.41)**	**1.37 (1.01–1.85)**
30–34 years	1.00	1.00
35–45 years	1.15 (0.80–1.67)	1.29 (0.90–1.83)
*Marital status*		
Married	1.00	1.00
Not married	1.12 (0.85–1.48)	**0.39 (0.28–0.53)**
*Maternal race*		
White, NH	1.00	1.00
Black, NH	1.31 (0.77–2.20)	**0.53 (0.29–0.96)**
Hispanic	**2.48 (1.36–4.49)**	1.38 (0.74–2.57)
Others	**5.36 (1.93–14.89)**	**4.03 (1.44–11.29)**
*Maternal education*		
Less than high school	**0.47 (0.26–0.83)**	**0.11 (0.06–0.22)**
High school	**0.55 (0.39–0.77)**	**0.20 (0.14–0.29)**
1–3 years of college	1.07 (0.79–1.45)	**0.46 (0.34–0.62)**
College graduate	1.00	1.00
*Income*		
<$20,000	**0.69 (0.49–0.96)**	**0.35 (0.24–0.49)**
$20,000–$49,999	0.83 (0.64–1.07)	**0.68 (0.53–0.88)**
≥$50,000	1.00	1.00
*Prepregnancy BMI*		
Underweight	0.73 (0.44–1.20)	**0.48 (0.28–0.81)**
Normal weight	1.00	1.00
Overweight	1.10 (0.81–1.48)	0.89 (0.66–1.20)
Obese	0.77 (0.58–1.03)	**0.58 (0.44–0.78)**
*Health insurance*		
No	1.04 (0.58–1.89)	1.32 (0.74–2.36)
Yes	1.00	1.00
*Postnatal WIC*		
No	1.00	1.00
Yes	**0.77 (0.61–0.97)**	**0.30 (0.24–0.38)**
*Prenatal WIC*		
No	1.00	1.00
Yes	**0.76 (0.60–0.97)**	**0.33 (0.25–0.42)**
*Mode of delivery*		
Vaginally, not induced	1.00	1.00
Vaginally, induced	1.05 (0.79–1.39)	**0.72 (0.54–0.95)**
Planned C-section	**0.62 (0.44–0.86)**	**0.60 (0.44–0.83)**
Unplanned or emergency C-section	1.18 (0.81–1.73)	**0.64 (0.43–0.95)**
*Breastfeeding intention*		
Breastfeed only	1.00	1.00
Formula or combination	**0.01 (0.01–0.27)**	**0.003 (0.001–0.007)**
*Smoked during pregnancy*		
No	1.00	1.00
Yes	**0.56 (0.41–0.76)**	**0.15 (0.10–0.22)**

OR = odds ratio; CI = confidence interval; NH = non-Hispanic; WIC = women; infants and children; BMI = body mass index; C-Section = Cesarean section. *Note*. Bold estimates are significant.

**Table 3:** Association between healthcare provider at birth and breastfeeding duration.

	Unadjusted COR (95% CI)		Parsimonious model^a^	
			AOR (95% CI)	
	Breastfed < 6 months	Breastfed ≥ 6 months	Breastfed < 6 months	Breastfed ≥ 6 months
Family	1.07 (0.64–1.77)	1.17 (0.70–1.94)	1.52 (0.82–2.79)	**2.04 (1.04**–**4.00)**
Physician/other Physicians				
Midwife/nurse midwife	**2.32 (1.22–4.42)**	**3.99 (2.12–7.49)**	1.43 (0.68–3.01)	**2.43 (1.12–5.25)**
Obstetrician	Reference			

COR = crude odd ratio; CI = confidence interval; AOR = adjusted odd ratio. *Note*. Never breastfeeding is the reference category. ^a^Parsimonious model controlling for marital status, education, race, income, age, prenatal WIC participation, postpartum WIC participation, mode of delivery, and breastfeeding intention.

**Table 4:** Association between healthcare provider at birth and exclusive breastfeeding duration.

	Unadjusted COR (95% CI)		Parsimonious model^a^	
			AOR (95% CI)	
	Breastfed < 6 months	Breastfed ≥ 6 months	Breastfed < 6 months	Breastfed ≥ 6 months
Family or other physicians	1.19 (0.71–1.99)	1.30 (0.54–3.16)	1.13 (0.65–1.98)	1.30 (0.51–3.31)
Midwife/nurse midwife	**4.01 (2.13–7.55)**	**9.96 (4.78–20.75)**	**3.11 (1.62–5.98)**	**6.65 (3.05–14.50)**
Obstetrician	Reference			

COR = crude odd ratio; CI = confidence interval; AOR = adjusted odd ratio. *Note*. Bold estimates are significant. Never breastfeeding is the reference category. ^a^Parsimonious model controlling for marital status, income, age, mode of delivery, prenatal WIC participation, and postpartum WIC participation.
